# Rapid Diagnosis of Ductal Carcinoma In Situ and Breast Cancer Based on Raman Spectroscopy of Serum Combined with Convolutional Neural Network

**DOI:** 10.3390/bioengineering10010065

**Published:** 2023-01-04

**Authors:** Xianglei Wang, Fei Xie, Yang Yang, Jin Zhao, Guohua Wu, Shu Wang

**Affiliations:** 1 School of Science, Beijing University of Posts and Telecommunications, Beijing 100876, China; 2 Department of Breast Center, Peking University People’s Hospital, Beijing 100044, China; 3 School of Electronic Engineering, Beijing University of Posts and Telecommunications, Beijing 100876, China

**Keywords:** breast cancer, ductal carcinoma in situ, Raman spectroscopy, serum, diagnosis

## Abstract

Ductal carcinoma in situ (DCIS) and breast cancer are common female breast diseases and pose a serious health threat to women. Early diagnosis of breast cancer and DCIS can help to develop targeted treatment plans in time. In this paper, we investigated the feasibility of using Raman spectroscopy combined with convolutional neural network (CNN) to discriminate between healthy volunteers, breast cancer and DCIS patients. Raman spectra were collected from the sera of 241 healthy volunteers, 463 breast cancer and 100 DCIS patients, and a total of 804 spectra were recorded. The pre-processed Raman spectra were used as the input of CNN to establish a model to classify the three different spectra. After using cross-validation to optimize its hyperparameters, the model’s final classification performance was assessed using an unknown test set. For comparison with other machine learning algorithms, we additionally built models using support vector machine (SVM), random forest (RF) and k-nearest neighbor (KNN) methods. The final accuracies for CNN, SVM, RF and KNN were 98.76%, 94.63%, 80.99% and 78.93%, respectively. The values for area under curve (AUC) were 0.999, 0.994, 0.931 and 0.900, respectively. Therefore, our study results demonstrate that CNN outperforms three traditional algorithms in terms of classification performance for Raman spectral data and can be a useful auxiliary diagnostic tool of breast cancer and DCIS.

## 1. Introduction

Breast cancer is a common malignancy in women worldwide and one of the main causes of cancer death for women. According to the International Agency for Research on Cancer (IARC), female breast cancer has become the most commonly diagnosed cancer in 2020, with an estimated 2.3 million new cases, accounting for 11.7% of new cancer cases, and also an estimated 680,000 deaths, accounting for 6.9% of cancer deaths [1]. In the recent decades, there has been a fast increase in the incidence of breast cancer worldwide, with developed nations seeing higher incidence rates and developing nations experiencing higher mortality rates [2,3]. In 2015, about 304,000 new cases of breast cancer occurred in Chinese women, representing 17.1% of all the incidence of all female malignancies, and about 70,000 deaths occurred, or 8.2% of all cancer deaths in women [4,5]. Breast carcinogenesis is a multi-step phenomenon, and one of the early cancers that occurs at a localized site is ductal carcinoma in situ (DCIS) [6]. Currently, DCIS accounts for approximately 20–30% of breast cancer diagnoses [7]. If not treated in time, a significant part of DCIS will progress to invasive carcinoma. Conversely, if treated properly, patients with DCIS will have a good prognosis, as death rate due to DCIS alone is less than 1–2% [6,7]. DCIS was rarely diagnosed until the 1980s, but its incidence increased rapidly as mammograms became more common. At present, there are more than 60,000 confirmed instances of DCIS in the US each year [8]. The data above show that the early diagnosis of DCIS and breast cancer is of great significance in implementing timely and effective medical treatments, which can greatly reduce the cancer mortality rate in women.

Currently, there are many clinical methods used to detect breast cancer and DCIS, including X-ray mammography, positron emission tomography (PET), magnetic resonance imaging (MRI) and ultrasound. X-ray mammography is the most commonly used screening tool. It is considered to be the gold standard for breast cancer diagnosis [9]. However, X-ray mammography has a higher rate of false positives and may also cause some harm to the patient’s body as it causes harmful ionizing radiation during screening [10,11,12]. The results of X-ray mammography generally require further methods to confirm. Histopathology is considered the gold standard for most cancer diagnoses by researchers and medical associations [13,14]. It mainly refers to the removal of a sample of suspicious tissue from a patient to make a tissue section and a thorough microscopic examination to determine whether the disease has occurred. This invasive method may cause physical trauma to the patient, and the pathological diagnosis is mainly based on the experience and criteria of the pathologist, factors which are highly subjective. Hence, it is of great clinical significance to develop a quick, highly sensitive and minimally invasive screening technology for breast cancer and DCIS.

Raman spectroscopy is a fast, sensitive and label-free optical analysis technique based on inelastic scattering of light. It is sensitive to slight structural changes in the molecule under study. Researchers can analyze a substance’s biochemical makeup and structural properties based on the intensity and location of the spectral peaks. Researchers have made extensive use of Raman spectroscopy to study biochemical changes in human serum and plasma. As the disease continues to progress, tumor metabolites can be found in the patient’s blood circulation, thus allowing the use of Raman spectroscopy to detect variations in the levels of substances such as proteins and lipids as the cancer progresses. In recent years, the use of Raman spectroscopy combined with machine learning for early screening of various diseases has been widely studied, and has been used for diseases such as oral cancer, nasopharyngeal cancer, lung cancer, brain cancer, and skin cancer [15]. For breast cancer and DCIS, a number of research groups have also conducted in-depth studies [16,17,18,19,20,21,22]. In the study of breast tissue, Rehman et al. used Raman spectroscopy to study DCIS and invasive ductal carcinoma (IDC), and found significant spectral differences between the various degrees of DCIS and IDC, which enabled objective distinction between DCIS and IDC grades using Raman spectroscopy [16]. Han et al. distinguished the Raman spectra of atypical ductal hyperplasia (ADH), DCIS, and IDC used support vector machine (SVM), and the SVM model achieved a classification accuracy of 74.39% [17]. Lazaro-Pacheco et al. successfully distinguished normal breast tissue from breast cancer tissue using principal component analysis-linear discriminant analysis (PCA-LDA), with 90% model sensitivity and 78% model specificity [18]. In the study of patient serum, Pichardo-Molina et al. classified Raman spectra from healthy and breast cancer serum samples using PCA-LDA, with a sensitivity of 97% and a specificity of 78% [19]. Nargis et al. compared the classification performance of surface-enhanced Raman spectroscopy (SERS) and Raman spectroscopy, and SERS combined with PLS-DA could better distinguish between normal people and breast cancer patients compared with Raman spectroscopy, with 90% model sensitivity and 98.4% model specificity [20]. Lin et al. analyzed the SERS spectra of normal and breast cancer serum proteins using PCA-LDA and partial least square-support vector machine (PLS-SVM) and showed that PLS-SVM had better diagnostic effect, with 94.67% model accuracy [21]. Furthermore, Zhang et al. obtained Raman spectra from cultured breast cancer cell lines, successfully distinguished normal cells from breast cancer cells using machine learning techniques, and further classified breast cancer subtypes [22]. The above studies show that Raman spectroscopy holds significant promise for DCIS and breast cancer screening. However, the sample size of the experiment is small and there is room for improvement in diagnostic accuracy. The use of breast tissue for spectral measurement is invasive, and the SERS experiment is relatively costly and complicated to perform. In addition, the process of culturing breast cell lines is long and complicated, which is not suitable for rapid diagnosis of breast cancer. Therefore, we tried to develop a simple, minimally invasive and highly accurate method for the rapid diagnosis of DCIS and breast cancer.

In this study, we used more serum samples (804 cases) than other studies to further study DCIS and breast cancer using the CNN algorithm. We directly used the normalized spectra as the input of CNN to establish a diagnostic model for DCIS and breast cancer, allowing the feature information in the original spectra to be fully utilized. The model finally achieved an accuracy of 98.76%. In addition, we compared the CNN algorithm with KNN, RF and SVM, and finally validated that the CNN model has higher diagnostic accuracy and better classification performance. To the best of our knowledge, this is the first study to simultaneously screen for breast cancer and DCIS using the CNN algorithm.

## 2. Materials and Methods

### 2.1. Sample Collection and Preparation

**Figure 1:**
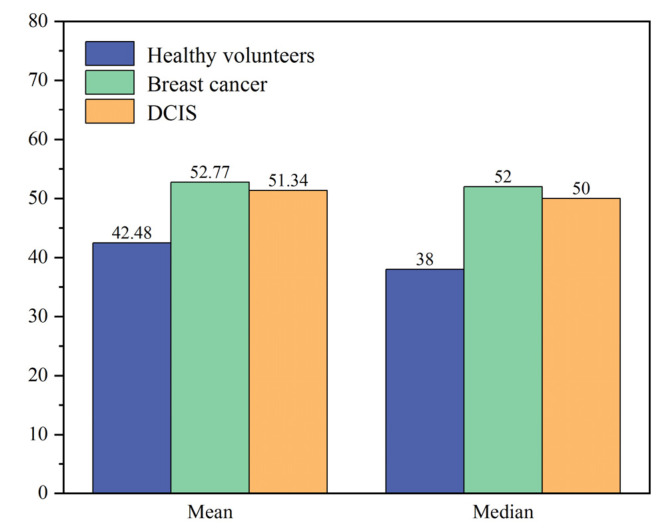
The age information of healthy volunteers, breast cancer and DCIS patients.

In this study, we obtained 804 serum samples, of which 241 were from healthy volunteers, 463 from breast cancer patients and 100 from DCIS patients. All of the above serum samples were provided by the Peking University People’s Hospital. The protocol and procedures of this study were approved by the Ethics Committee of Peking University People’s Hospital (approval no.: 2021PHB169-001). All participants signed an informed consent form. Two milliliters of morning fasting venous blood was collected from each participant. All blood samples were obtained from the median cubital vein. For breast cancer patients, blood samples were collected in the morning of the day before surgery. The collected blood samples were placed at room temperature for 2 h and then centrifuged at 4 °C, 3000 RPM for 10 min. Serum was obtained by extracting the upper clarified layer. The obtained serum was poured into different tubes and stored in a refrigerator at −80 °C prior to Raman spectroscopy measurements. Each sample tub was labeled with a barcode corresponding to the corresponding participant. Blood collection and serum preparation are performed by highly trained professionals. Aseptic principle and the “Standard Operating Procedures of the Biobank of Peking University People’s Hospital” were strictly observed throughout the process of blood collection and serum extraction. Personal information of participant will was kept confidential. The age distribution of all participants in this study is shown in Figure 1. All subjects were female. Among the healthy volunteers, the age ranged from 21 to 85 years, with a mean age of 42.48 years and a median age of 38 years. Among breast cancer patients, the age ranged from 26 to 87 years, with a mean age of 52.77 years and a median age of 52 years. Among DCIS patients, the age ranged from 22 to 80 years, with a mean age of 51.34 years and a median age of 50 years. It can be seen that the mean and median ages of breast cancer patients and DCIS patients were close, with both showing significant differences from the age distribution of healthy volunteers.

### 2.2. Raman Spectral Measurements

A 10 µL drop of serum was drawn from each sample tube onto aluminum foil. The B&WTEK BWS465-532S Raman spectrometer (USA) was used to collect the Raman spectra, with an excitation wavelength of 532 nm, a laser power of 8 mW and a spectrometer resolution of 4.5 cm^−1^. The five spectra were collected from five different locations on the surface of each sample and their average spectra were used for subsequent analysis. Each spectrum’s exposure integration duration was 10 s, and the spectra of all samples were collected within 400 to 4000 cm^−1^.

### 2.3. Spectra Preprocessing Method

The Raman spectra of biological samples generally contain noise and background baselines, and the pure Raman spectra can be obtained after removing them. The Savitzky–Golay method [23] was used to smooth out all of the spectra, and this algorithm can remove noise while leaving the signal’s width and shape unchanged. After denoising, the background baseline was filtered from the spectral data by adaptive iteratively reweighted penalized least squares (airPLS). The baseline corrected spectra were subjected to max–min scaling to mitigate the effect of different spectral peak variations. Therefore, we consider only the relative Raman intensity in the subsequent analysis.

### 2.4. Classification Model

#### 2.4.1. K-Nearest Neighbor (KNN)

The KNN algorithm is a common supervised classification algorithm. For a new input sample, the K-nearest samples to it are selected by calculating its distance from all known category samples. According to the principle of minority obeying majority, it is classified as that category which is in the majority in K samples. The choice of K value is crucial. If a small K value is selected, other samples adjacent to the K samples will bring noise to the model and cause model overfitting. If a larger K value is selected, the samples at a distance will also affect the classification results and cause prediction errors [24]. In this study, we find the optimal K-values through the cross-validation on the training set.

#### 2.4.2. Random Forest (RF)

The RF algorithm is an integrated learning algorithm, consisting of multiple independent decision trees. Each decision tree is built by randomly sampling the training data and then by completely splitting the sampled data. When new examples are fed into the RF model, each decision tree sorts the input examples according to the features it has selected [25]. Finally, the output of each decision tree is combined, and the final decision is obtained based on majority voting. Since each decision tree selects a partial training sample and chooses features randomly; this can avoid overfitting to some extent [24]. In the RF model building process, we optimized the predictive power of the model by adjusting the number and depth of trees through cross-validation.

#### 2.4.3. Support Vector Machine (SVM)

Support vector machine (SVM) is a supervised classification algorithm based on statistical learning theory [26]. The fundamental principle of the SVM algorithm is to partition the data set, using an optimal hyperplane to maximize the geometric separation between the hyperplane and the data points on its sides, which can be formalized as a convex quadratic optimization problem. For linearly indistinguishable data sets, SVM can use kernel functions to map them to a higher dimensional space and make them linearly distinguishable. It is considered better than traditional linear classification methods [27]. The kernel function and the penalty factor C are the two most critical parameters of the SVM [28]. In this study, we choose the most commonly used radial basis function (RBF) as the kernel function and find the optimal parameters by grid search.

#### 2.4.4. Convolutional Neural Networks (CNN)

**Figure 2:**
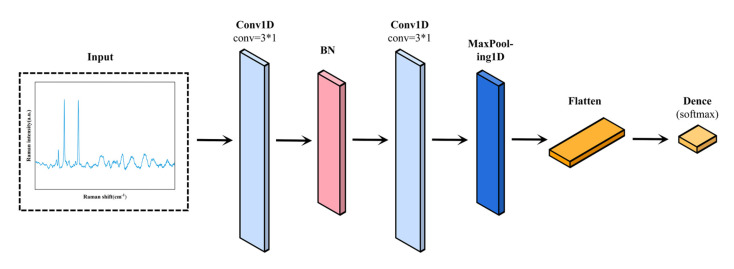
One-dimensional CNN network structure.

CNN is a crucial component of deep learning algorithms, with features such as local connectivity and weight sharing which can effectively solve the problems of many parameters and the easy overfitting of traditional neural networks. Natural language processing and image categorization are two areas where it has seen extensive usage [29]. We constructed a one-dimensional CNN model to categorize Raman spectra. Figure 2 shows the structure of our CNN model. The Raman spectral data input to the model is the one-dimensional vector of length 1698. The hidden layer contains a convolutional layer, a batch normalization layer and a pooling layer. We use two convolutional layers and one pooling layer to extract Raman spectral characteristics. The first convolution layer contains 8 output channels, and 8 feature mappings are extracted from the Raman spectral data using a convolution kernel of length 3. The number of channels in the second convolution layer is doubled to 16, and more feature mappings are extracted from the previous output data. The shared convolution kernel can effectively reduce computational complexity and keep the classifier stable during the spatial transformation [30]. Following the second convolutional layer is a max-pooling layer with a pool size of 1 × 3. The pooling layer can perform feature compression to extract the main features and can also avoid overfitting to a certain extent. We also added a batch normalization layer between the convolutional layers, a development which can speed up the network training and prevent the overfitting of the model. After that, these extracted features were input into the fully connected layer and the output layer with the number of nodes at 9024 and 3, respectively, which could be viewed as a classifier. The output function was softmax, which could calculate the probability of each sample belonging to each category separately and use the category with the highest probability as the category of the sample for classification. During the training of the CNN model, we used cross-entropy loss combined with gradient descent to continuously update the CNN’s weights and biases, and optimized the model hyperparameters by a 5-fold cross-validation of the training set. Finally, an unknown test set was fed into the trained model to evaluate the classification performance.

In this study, all data analysis and model building were done through Python 3.7. Among them, KNN, RF and SVM models were built by Sklearn in Python 3.7, and CNN was built by Keras and Tensorflow in Python 3.7.

## 3. Results and Discussions

### 3.1. Spectral Data Analysis

**Figure 3:**
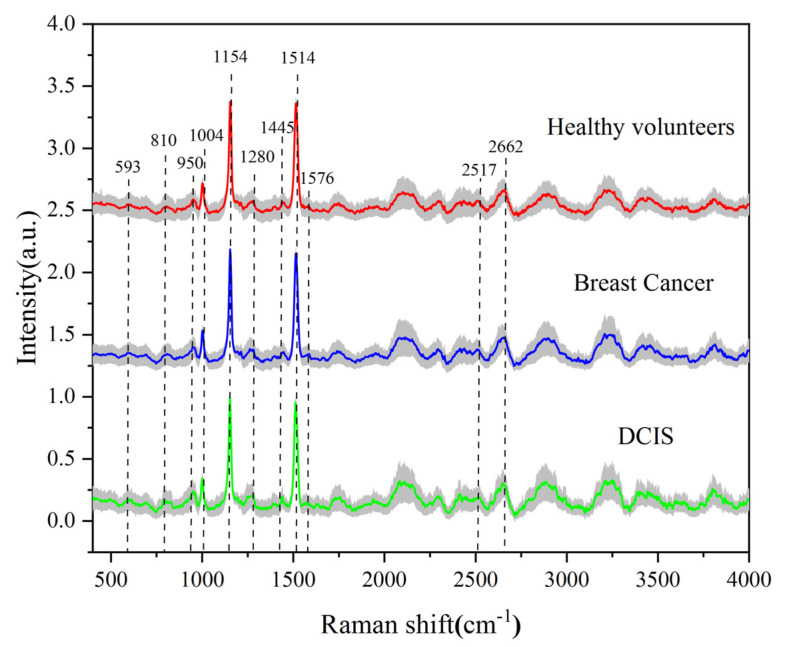
Comparison of normalized mean Raman spectra of serum sample for three groups: (1) Healthy volunteers (n = 241), (2) Breast cancer patients (n = 463) and (3) DCIS patients (n = 100). The shaded area is the standard deviation of the spectra.

**Figure 4:**
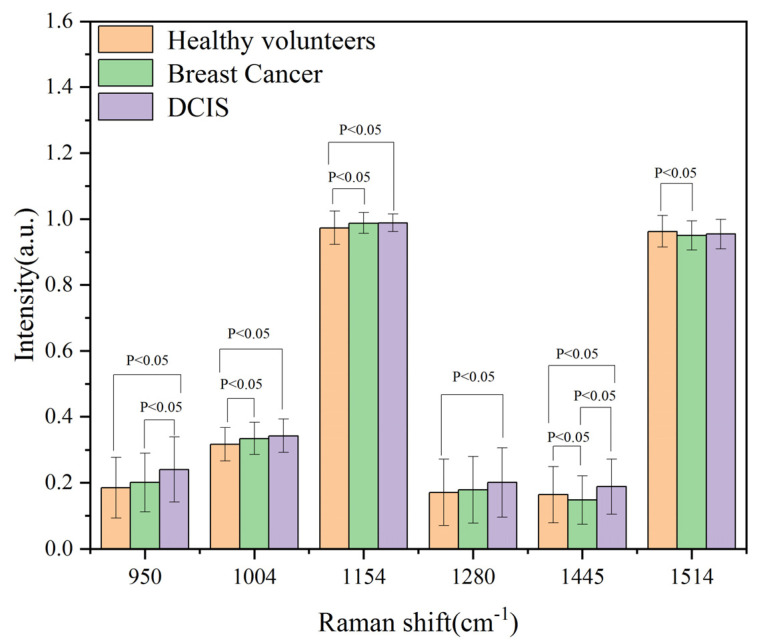
Mean intensities and standard deviations of the main Raman peaks.

The Raman spectra of 241 healthy volunteers, 463 breast cancer patients and 100 DCIS patients were denoised, baseline-corrected and normalized. Figure 3 shows their normalized average spectra. The shaded part of the figure shows the standard deviation of their respective mean spectra. In order to show the three average spectra more visually, we have shifted the spectral lines appropriately. It can be seen that the shapes of the three average spectra are very similar, and the positions of their characteristic peaks are almost the same. This indicates that the biomolecular components contained in the three types of serum samples are similar. The Raman peaks in healthy volunteers, breast cancer and DCIS patients were prominent at 950 cm^−1^, 1004 cm^−1^, 1154 cm^−1^, 1280 cm^−1^, 1445 cm^−1^, 1514 cm^−1^, 2517 cm^−1^, and 2662 cm^−1^, with the strongest signals given at 1154 cm^−1^ and 1514 cm^−1^. The mean intensities and standard deviations at 950 cm^−1^, 1004 cm^−1^, 1154 cm^−1^, 1280 cm^−1^, 1445 cm^−1^ and 1514 cm^−1^ are given in Figure 4, showing the significant differences between the three sample groups. Table 1 lists the molecular assignment of the Raman peaks of normal, breast cancer and DCIS sera [31,32,33,34,35,36,37]. The peak at around 593 cm^−1^ is assigned to ascorbic acid and amide-VI. The peak at around 810 cm^−1^ is assigned to C-C-O stretching vibration of L-serine and glutathione. The peak at around 950 cm^−1^ is assigned to C-C stretching vibration of proline and valine. The peaks at around 1004 and 1576 cm^−1^ are assigned to C-C symmetric stretch and C-C bending of Phenylalanine, respectively. The peak at around 1280 cm^−1^ is assigned to CH_2_ wagging vibration from nucleic acid. The peaks at around 2517 and 2662 cm^−1^ are assigned to O-H stretching of proteins. The peak at around 1445 cm^−1^ is assigned to CH_2_ bending vibration of collagen and phospholipids, and this peak has diagnostic significance in medical research [38]. Finally, the relatively sharp and enhanced peaks of 1154 and 1514 cm^−1^ are assigned to C-C stretch. These originate from the Raman signals of β-carotene.

### 3.2. CNN Model Establishment and Classification

**Figure 5:**
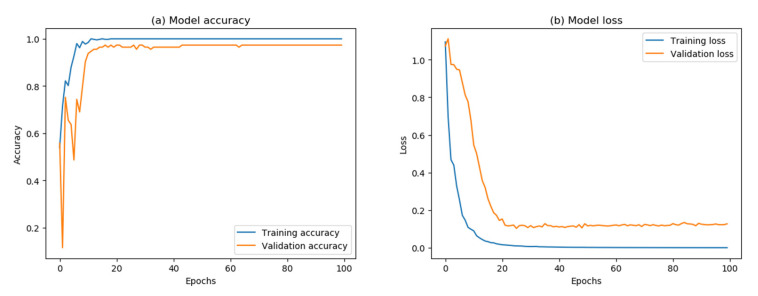
(**a**) Accuracy curves of training set and validation set. (**b**) Loss curves of training set and validation set.

First, we divided all the spectra after they had been pre-processed into a training set and a test set at the ratio of 7:3 randomly. A total of 562 training samples and 242 test samples were obtained. Table 2 shows the details of the division. Since the default parameters of CNN are not optimal in general, we need to adjust the hyperparameters to make the model achieve the best classification effect. In this study, we chose to adjust the batch size, learning rate, epoch, activation function and optimizer, and used categorical_crossentropy as the loss function. The 5-fold cross-validation of the training set combined, with a grid search, was used to find the combination of hyperparameters that gives the highest cross-validation accuracy. Table 3 shows the optimal hyperparameter results. After that, we trained the model using the training set and the optimized hyperparameters to obtain the final weights and biases. Figure 5 shows the accuracy and loss of the CNN on the training and validation sets. Before the 20th epoch, the accuracy of training and validation sets fluctuated and slowly increased, while the loss also decreased. With the constant iteration of network parameters, both accuracy and loss converged to a small range after the 40th epoch. Finally, we used an unknown test set to evaluate the final generalization ability of the trained CNN model. The test set’s final classification accuracy was 98.76%, and 239 out of the 242 test samples were correctly classified.

**Figure 6:**
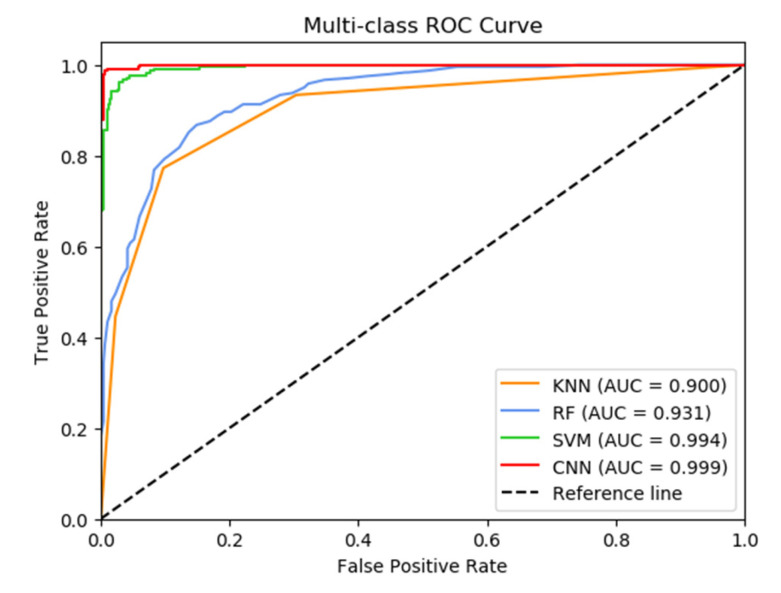
The ROC curves of KNN, RF, SVM and CNN models.

In order to contrast the classification effect of CNN and other three traditional machine learning algorithms, we used the same training and testing sets as the CNN model to build the model with KNN, RF and SVM algorithms. After testing the models using the test set, Table 4 shows the confusion matrix [39] for the KNN, RF, SVM and CNN models. As can be seen from Table 4, among the three traditional machine learning algorithms, the highest accuracy is 94.63% (SVM) and the lowest accuracy is 78.93% (KNN). The accuracy of all three traditional algorithms is lower than that of the CNN method. The numbers of misdiagnoses of the KNN, RF, SVM and CNN models are 51, 46, 13 and 3, respectively, indicating that the misdiagnosis rate of CNN is the lowest. Next, we further confirmed this result with the ROC curve. The area under the ROC curve is known as the area under curve (AUC). The closer the model’s AUC value is to 1, the better the prediction performance [40]. Figure 6 shows the ROC curves for the four diagnostic models. The AUC for the KNN, RF, SVM and CNN models are 0.900, 0.931, 0.994 and 0.999, respectively. It is clear that the CNN has the highest AUC value, further demonstrating the model’s strong classification performance.

Moreover, we used the same training and test sets as before to group the data two-by-two according to sample categories. The KNN, RF, SVM and CNN models were used to perform binary classification on them. Table 5 shows the binary classification results of the four algorithms. For healthy people and breast cancer groups, the RF model has the lowest accuracy (79.72%) and the CNN model has the highest accuracy (98.11%). For healthy people and DCIS groups, the KNN model has the lowest accuracy (90.29%), both SVM and CNN models have excellent classification results, and they both achieve the 100% classification accuracy. For breast cancer and DCIS groups, the accuracy of the KNN and the RF model are close, 91.72% and 92.90%, respectively. However, their sensitivities are relatively low, 73.33% and 60.00%, respectively. The classification performance of the SVM and CNN models is still excellent and the accuracy are all 100.00%. The above results show that, for the binary classification of serum Raman spectra, the CNN model has the highest sensitivity, specificity and accuracy, reflecting the excellent classification performance of CNN models.

The findings of the aforementioned study indicate that combining Raman spectroscopy with CNN can well distinguish healthy people, DCIS and breast cancer patients. As a result, this method has a good application prospect in the early detection of DCIS and breast cancer.

## 4. Conclusions

In this paper, we used Raman spectroscopy, combined with CNN, to study DCIS and breast cancer. We used serum Raman spectra collected from healthy people, DCIS and breast cancer patients to establish a one-dimensional CNN diagnostic model and compared it with traditional KNN, RF and SVM algorithms. Finally, the results showed that the diagnostic accuracy of the CNN method for healthy people, DCIS and breast cancer reached 98.76%. Additionally, the AUC was 0.999. All of these scores were higher than those of the three traditional machine learning algorithms. This exploratory study demonstrates that the serum Raman spectroscopy, combined with CNN, has great potential for early, fast, accurate, and minimally invasive screening of DCIS and breast cancer. It has good application prospects, and we will continue to study it in depth.

## Figures and Tables

**Table 1:** The assignments of Raman peak in the serum spectra.

Peaks	Major Assignments	Ref
593 cm^−1^	Ascorbic acid, amide-VI	Chen et al. [31]
810 cm^−1^	L-serine, glutathione: C-C-O stretching vibration	Wu et al. [32]
950 cm^−1^	α-Helix, proline, valine: C-C stretching vibration	Xia et al. [33]
1004 cm^−1^	Phenylalanine: C-C symmetric stretch	Zhang et al. [34]
1154 cm^−1^	β-carotene: C-C stretch	Ullah et al. [35]
1280 cm^−1^	Nucleic acid: Amide III & CH_2_ wagging vibration	Bahreini et al. [36]
1445 cm^−1^	Collagen, phospholipids: CH_2_ bending vibration	Zhang et al. [34]
1514 cm^−1^	β-carotene: C-C stretch	Ullah et al. [35]
1576 cm^−1^	Phenylalanine, acetoacetate, riboflavin:C-C bending	Li et al. [37]
2517 cm^−1^	Proteins: O-H stretch	Bahreini et al. [36]
2662 cm^−1^	Proteins: O-H stretch	Bahreini et al. [36]

**Table 2:** Division of train set and test set.

Samples	Normal	Breast Cancer	DCIS	Total
Training set	168	324	70	562
Test set	73	139	30	242
Total	241	463	100	804

**Table 3:** Optimal parameters of the CNN model.

Parameters	Batch Size	Epoch	Learning Rate	Optimizer	Activation	Loss	Cross-Validation Accuracy (%)
Value or option	16	100	0.001	Adam	Tanh	Categorical_ crossentropy	96.09

**Table 4:** Confusion matrix for the KNN, RF, SVM and CNN algorithms.

	**KNN**			**RF**			**SVM**			**CNN**		
	HC	BC	DCIS	HC	BC	DCIS	HC	BC	DCIS	HC	BC	DCIS
HC	47	24	2	43	30	0	67	6	0	72	1	0
BC	9	127	3	5	134	0	7	132	0	2	137	0
DCIS	2	11	17	1	10	19	0	0	30	0	0	30
Acc	78.93%			80.99%			94.63%			98.76%		

HC: Healthy controls; BC: Breast cancer; DCIS: Ductal carcinoma in situ; Acc: Diagnostic accuracy.

**Table 5:** Confusion matrix for binary classification of four algorithms.

	**KNN**		**RF**		**SVM**		**CNN**	
	HC	BC	HC	BC	HC	BC	HC	BC
HC	53	20	50	23	69	4	71	2
BC	16	123	20	119	8	131	2	137
Sensitivity	88.49%		85.61%		94.25%		98.56%	
Specificity	72.60%		68.49%		94.52%		97.26%	
Accuracy	83.02%		79.72%		94.34%		98.11%	
	**KNN**		**RF**		**SVM**		**CNN**	
	HC	DCIS	HC	DCIS	HC	DCIS	HC	DCIS
HC	69	4	72	1	73	0	73	0
DCIS	6	24	5	25	0	30	0	30
Sensitivity	80.00%		83.33%		100.00%		100.00%	
Specificity	94.52%		98.63%		100.00%		100.00%	
Accuracy	90.29%		94.18%		100.00%		100.00%	
	**KNN**		**RF**		**SVM**		**CNN**	
	BC	DCIS	BC	DCIS	BC	DCIS	BC	DCIS
BC	133	6	139	0	139	0	139	0
DCIS	8	22	12	18	0	30	0	30
Sensitivity	73.33%		60.00%		100.00%		100.00%	
Specificity	95.68%		100.00%		100.00%		100.00%	
Accuracy	91.72%		92.90%		100.00%		100.00%	

HC: Healthy controls; BC: Breast cancer; DCIS: Ductal carcinoma in situ.

## Data Availability

Data cannot be shared due to privacy or ethical restrictions.
